# Co-production of Xylooligosaccharides and Xylose From Poplar Sawdust by Recombinant Endo-1,4-β-Xylanase and β-Xylosidase Mixture Hydrolysis

**DOI:** 10.3389/fbioe.2020.637397

**Published:** 2021-02-01

**Authors:** Qi Li, Yunpeng Jiang, Xinyi Tong, Linguo Zhao, Jianjun Pei

**Affiliations:** ^1^Jiangsu Co-Innovation Center for Efficient Processing and Utilization of Forest Products, Nanjing Forestry University, Nanjing, China; ^2^College of Chemical Engineering, Nanjing Forestry University, Nanjing, China; ^3^Co-innovation Center for Sustainable Forestry in Southern China, Nanjing Forestry University, Nanjing, China

**Keywords:** poplar sawdust, xylooligosaccharide, synergetic enzymolysis, endo-1, 4-β-xylanase, β-xylosidase

## Abstract

As is well-known, endo-1,4-β-xylanase and β-xylosidase are the rate-limiting enzymes in the degradation of xylan (the major hemicellulosic component), main functions of which are cleavaging xylan to release xylooligosaccharides (XOS) and xylose that these two compounds have important application value in fuel, food, and other industries. This study focuses on enzymatic hydrolysis of poplar sawdust xylan for production of XOS and xylose by a GH11 endo-1,4-β-xylanase MxynB-8 and a GH39 β-xylosidase Xln-DT. MxynB-8 showed excellent ability to hydrolyze hemicellulose of broadleaf plants, such as poplar. Under optimized conditions (50°C, pH 6.0, dosage of 500 U/g, substrate concentration of 2 mg/mL), the final XOS yield was 85.5%, and the content of XOS_2−3_ reached 93.9% after 18 h. The enzymatic efficiency by MxynB-8 based on the poplar sawdust xylan in the raw material was 30.5%. Xln-DT showed excellent xylose/glucose/arabinose tolerance, which is applied as a candidate to apply in degradation of hemicellulose. In addition, the process and enzymatic mode of poplar sawdust xylan with MxynB-8 and Xln-DT were investigated. The results showed that the enzymatic hydrolysis yield of poplar sawdust xylan was improved by adding Xln-DT, and a xylose-rich hydrolysate could be obtained at high purity, with the xylose yield of 89.9%. The enzymatic hydrolysis yield was higher (32.2%) by using MxynB-8 and Xln-DT together. This study provides a deep understanding of double-enzyme synergetic enzymolysis of wood polysaccharides to valuable products.

## Introduction

Hemicellulose is a structural polysaccharide that constitutes agricultural and forestry plant cell wall, which is often cross-linked with cellulose and lignin to form lignocellulose (Yang et al., 2020). As one of the richest and cheapest renewable resources on earth, hemicellulose is mainly composed of pentose (d-xylose, l-arabinose) and (or) hexose (d-glucose, d-mannose, d-galactose) and other monosaccharide groups (xylose is the most abundant) (Lahtinen et al., 2019; Qi et al., 2020). The side chain may also contain a small amount of glucuronic acid (d-glucuronic acid, 4-O-methyl-d-glucuronic acid, and d-galacturonic acid) groups (Sun et al., 2013). Therefore, hemicellulose has tremendous latent capacity in the production of biofuels, feeds, and other chemicals, which contribute to the development of a circular economy (Ordomsky et al., 2015; Wang et al., 2020). Poplar is a kind of fast-growing and high-yield tree species, which has become an important industrial raw material and is used for civil construction, pulp and paper making, wood board processing, etc. (Christersson, 2008; Kiara et al., 2014; Dong et al., 2020). With the processing and utilization of poplar wood resources, a large number of wood residues and sawdust have been produced. These poplar sawdust residues contain 20–35% hemicellulose, which xylosyl group accounted for 30–40% of total sugar (Yan et al., 2015). Obviously, poplar sawdust has become one of the potential raw materials for efficient utilization of hemicellulose.

Xylan, as the most abundant hemicellulose in nature, is linked with xylopyranosyl group through β-1,4-glycosidic bond and may be replaced by glucuronic acid, arabinose, and acetic acid at the C2 and C3 positions of xylose (Li et al., 2020b). The main way to produce xylooligosaccharides (XOS) and xylose is from the degradation of xylan. XOS can selectively promote the proliferation of bifidobacteria and other probiotics, promote calcium absorption, and resist dental caries in human intestinal tract, among which the most effective components are xylobiose, xylotriose, and xylotetraose (Vázquez et al., 2000; Hsu et al., 2004). Moreover, compared with other functional oligosaccharides, XOS have good thermal stability and are not easy to degrade in the range of pH 2.0–7.0. In the biomass industry, XOS and xylose can be converted into ethanol, furfural, and other valuable fuels or chemicals (Zhang et al., 2015; Li et al., 2020a; Wang and Fang, 2020). At present, various pretreatment methods, such as self-hydrolysis, enzymatic hydrolysis, acid hydrolysis, microwave-assisted method, and enzymatic acid hydrolysis can be used to degrade xylan to obtain XOS with different polymerization degrees (Wei et al., 2018; Li et al., 2019b; Wen et al., 2019; Jun et al., 2020). Among these methods, through the self-hydrolysis of lignocellulose, the yield is low, and the by-products are multiple; the acid hydrolysis method using sulfuric acid or hydrochloric acid brings great burden to the environment. Compared with them, enzymatic hydrolysis is a powerful tool to convert hemicellulose into value-added products in an environmental friendly way because of its mild reaction conditions, being pollution-free, and high conversion efficiency (Menezes et al., 2009; Li et al., 2019a). Enzymatic degradation of xylan into XOS, disaccharides, monosaccharides, and other simpler components usually requires the combined use of a variety of relatively specific enzymes. Endo-1,4-β-xylanase and β-xylosidase are important components of enzyme cocktails for hydrolysis of xylan. Endo-1,4-β-xylanase can cut off the β-1,4-xylcoside bonds in the main chain of xylan randomly to produce XOS, but not produce too many xyloses. Subsequently, β-xylosidase can continue to degrade the terminal, non-reducing β-d-xylosyl residues to release xylose, thus alleviating the inhibition of endo-1,4-β-xylanase by XOS (Blake et al., 2018). The synergy among endo-1,4-β-xylanase, β-xylosidase, and other side-chain enzymes leads to that the hydrolysis efficiency of xylan can be significantly accelerated, thus reducing the cost of enzyme. Zhuo et al. (2018) reported a xylanase and β-xylosidase from white rot fungi and their application in synergistic hydrolysis of lignocellulose, in which the two enzymes could efficiently improve the hydrolysis rate of cornstalk pretreated by sodium hydroxide. Jamaldheen et al. (2019) optimized two-step enzymatic saccharification of hemicellulose part of the pretreated finger millet straw for production of xylose using endo-1,4-β-xylanase and exo-1,4-β-xylosidase with the percentage conversion yield of 24.7%.

From the source, endo-1,4-β-xylanase and β-xylosidase can be produced by many organisms, such as fungi, bacteria, plant, and archaea (Li et al., 2017, 2020c; Shi et al., 2018; Zhang et al., 2019b). Nowadays, the hot spot of research is still microbial source endo-1,4-β-xylanases and β-xylosidases. According to the amino acid sequence homology comparison, endo-1,4-β-xylanase was distributed in the glycoside hydrolases GH5, GH8, GH10, GH11, and GH43 (Vincent et al., 2014). Among them, endo-1,4-β-xylanases from GH11 family are widely used in lignocellulose degradation and food additives because of their high specificity, low molecular weight, and high catalytic efficiency (Juturu and Wu, 2012). At present, endo-1,4-β-xylanases from GH11 family are mainly distributed in *Aspergillus, Trichoderma*, and *Penicillium* (Collins et al., 2010). Generally, the expression level of endo-1,4-β-xylanases from filamentous fungi was much higher than that of bacterial endo-1,4-β-xylanases (Li et al., 2012). However, the optimum temperature and temperature stability of filamentous fungi endo-1,4-β-xylanases are far less than those of bacteria (Li et al., 2017). Merely, the thermostability of endo-1,4-β-xylanases from filamentous fungi can be improved by genetic engineering; thereby, the application scope of the filamentous fungi endo-1,4-β-xylanases is greatly extended (Li et al., 2019c). All the β-xylosidases are mainly divided into GH1, GH3, GH30, GH39, GH43, GH52, GH54, and GH120 (Rohman et al., 2019). However, most β-xylosidases, especially the β-xylosidases belonging to GH3, are sensitive and inhibited to xylose by feedback, which limits its practical application. The reported *K*_*i*_ values of β-xylosidases from *Penicillium oxalicum, Humicola insolens* Y1, *Thermotoga petrophila*, and *Dictyoglomus turgidum* were in the range of 1.857–28.09 mM (Yang et al., 2014; Ye et al., 2017; Zhang et al., 2019b; Li et al., 2020c). Along with a growing number of β-xylosidases discovered, β-xylosidases from GH39 family with high xylose tolerance (*K*_*i*_values of 210–3,300 mM) are gradually recognized (Corrêa et al., 2012; Bhalla et al., 2014; Li et al., 2018). Thus, the application efficiency of GH39 family β-xylosidases in xylan hydrolysis was greatly improved.

In this article, a GH11 family endo-1,4-β-xylanase MxynB-G116C-Y135C-S58H-D76R-N28H-N29d-Y45M-N47L (MxynB-8) from *Aspergillus niger* NL-1, which has been modified by heat resistance (Li et al., 2019c), and a GH39 family β-xylosidase Xln-DT from *Dictyoglomus thermophilum* (Li et al., 2018) were used to hydrolyze poplar sawdust. At the same time, the enzymolysis conditions were optimized. In addition, the effect and mode of enzymatic hydrolysis of poplar sawdust xylan were also studied by MxynB-8 and Xln-DT. All the results will lay a foundation for the high-value application of poplar sawdust xylan and determine its potential applicability to lignocellulosic bioethanol production.

## Materials and Methods

### Materials and Reagents

The recombinant plasmids pTrc-99a-*mxynB*-G116C-Y135C-S58H-D76R-N28H-N29d-Y45M-N47L, pET-20b-*xynB-DT*, pET-28a-*xln-DT*, and pET-20b-*dt-xyl3* were constructed and preserved by Microbial Technology Research Laboratory (Nanjing Forestry University, China). *Escherichia coli* BL21 (DE3) was the expression host, which were preserved in Microbial Technology Research Laboratory. The bacterial strains were grown overnight at 37°C in Luria–Bertani (LB) medium containing kanamycin or ampicillin (100 μg/mL).

Poplar sawdust was purchased from Fukang wood processing plant (Jiangsu, China). The analysis of chemical components of poplar sawdust was carried out according to the National Renewable Energy Laboratory Standard (Abbas et al., 2020). The moisture content of milled poplar sawdust was ~9.8%. The results showed that the cellulose content of poplar sawdust was 38.91%, followed by hemicellulose (26.93%) and lignin (18.22%). Poplar sawdust xylan was extracted by alkali method. The optimum extraction conditions were as follows: the mass fraction of alkali solution was 10%, the ratio of solid to liquid was 1:10 (wt/vol), and the temperature was 120°C, whereas the yield of poplar xylan could reach 20.7% after 3 h. According to infrared radiation, acid hydrolysis, and high-performance liquid chromatography (HPLC) results, it showed that xylose was the main component of poplar sawdust xylan, accounting for 88.69%, and containing 4.76% cellobiose and 6.62% glucose (Supplementary Figure 1). The structure of xylan was mainly composed of β-configuration pyran ring (Supplementary Figure 2).

The substrate *p-*nitrophenyl-β-d-xylopyranoside (*p*NPX), *p*NP-β-d-glucoside (*p*NPG), *o*NP-β-d-glucoside (*o*NPG), *p-*nitrophenyl-β-d-galactopyranoside (*p*NPGal), *p-*nitrophenyl-α-l-rhamnopyranoside (*p*NPR), *p*NP-α-l-arabinofuranoside (*p*NPA), beechwood xylan, birchwood xylan, oat spelt xylan, d-glucose, d-xylose, d-galactose, d-mannose, and l-arabinose were purchased from Sigma–Aldrich (USA). Xylobiose (X_2_), xylotriose (X_3_), xylotetraose (X_4_), xylopentaose (X_5_), and xylohexaose (X6) were purchased from Megazyme (Ireland), and were used as standards. All other conventional chemical reagents were of analytic grade and obtained from general commercial sources.

### Protein Expression, Purification, Sodium Dodecyl Sulfate–Polyacrylamide Gel Electrophoresis Assay, and Enzyme Assay

The four recombinant expression plasmids were transformed into *E. coli* BL21 (DE3) and grown in LB kanamycin or ampicillin (100 μg/mL) medium at 37°C overnight. Then, the transformants were induced to express the recombinant MxynB-8, XynB-DT, Xln-DT, and Dt-xyl3, respectively, to an absorbance at OD_600_ ~0.8 before being induced with 0.01 mM of isopropyl-β-d-thiogalactopyranoside (IPTG), and then the bacteria were further incubated at 28°C for 6–14 h. Each cultures were harvested by centrifugation at 10,000 revolutions/min for 10 min at 4°C, and then the pellets were resuspended in 20 mM Tris-HCl buffer (pH 7.9) and broken up by ultrasonication (Ultrasonic Cell Pulverizer, China). The cell lysate after ultrasound was centrifuged at 10,000 revolutions/min for 20 min at 4°C to remove inclusion bodies. The crude endo-1,4-β-xylanase MxynB-8, XynB-DT and β-xylosidase Xln-DT, Dt-xyl3 were purified by an immobilized metal affinity column (Novagen, USA). Finally, the pure endo-1,4-β-xylanase and β-xylosidase proteins were collected by eluting with 1 M imidazole, 0.5 M NaCl, and 200 mM Tris-HCl buffer (pH 7.9), respectively.

Purities of the target proteins were performed by sodium dodecyl sulfate–polyacrylamide gel electrophoresis (SDS-PAGE) on a 12.5% gel, visualized by staining with Coomassie brilliant blue R-250, and the protein bands were analyzed by density scanning with Bio-Rad Analysis System (USA). The purified protein concentrations of the samples were determined by the Bradford method with bovine serum albumin as a standard (Chen et al., 2015).

Endo-1,4-β-xylanase activity was measured with a 3,5-dinitrosalicylic acid assay by using 1% solubilized beechwood xylan as a substrate in a 200 μL of reaction mixture containing 100 μL of 50 mM sodium citrate buffer (pH 6.0), 50 μL of beechwood xylan, and 50 μL of purified enzyme at the optimal temperature for 30 min. The activity of the enzyme without preincubation was defined as 100%. One unit of endo-1,4-β-xylanase activity was defined as the amount of enzyme that releases 1 μmol of reducing xylose from the substrate solution per minute. All measurements were performed in triplicate.

β-Xylosidase activity was assayed using *p*NPX as a substrate in a 200 μL of reaction mixture containing 180 μL of 50 mM sodium phosphate buffer (pH 6.0), 10 μL of 20 mM substrate *p*NPX, and 10 μL of purified enzyme. After incubation at the optimal temperature for 5 min, the reaction was stopped by adding 600 μL of 1 M Na_2_CO_3_. The absorbance of the released *p*NP was immediately measured at 405 nm. One unit of β-xylosidase activity was defined as the amount of enzyme releasing 1 μmol of *p*NP from the substrate solution per minute under the assay conditions. All measurements were performed in triplicate.

The substrate specific activities and kinetic constant (*K*_m_ and *V*_max_) of the purified enzymes Xln-DT and Dt-xyl3 were tested by using *p*NPX, *p*NPG, *o*NPG, *p*NPGal, *p*NPR, and *p*NPA ranging from 0.2 to 8 mM under standard reaction conditions. The effects of various xylose, arabinose, and glucose concentrations (20, 50, 100, and 500 mM) on the β-xylosidase activity of purified enzyme Xln-DT and Dt-xyl3 were measured. Production of xylose and glucose from xylobiose and cellobiose (0.2, 0.5, 1.0, 2.0, 3.0, 5.0, 8.0, and 10.0 g/L), respectively, by the purified enzyme Xln-DT and Dt-xyl3 was examined. The activity of the enzyme without the sugar was defined as 100%. All experiments were performed in triplicate.

### Enzymatic Hydrolysis of Beechwood, Birchwood, Oat Spelt, and Poplar Sawdust Xylan

Endo-1,4-β-xylanase MxynB-8 and XynB-DT were used by hydrolysis of 2 mg/mL beechwood, birchwood, oat spelt, and poplar sawdust xylan, respectively, in 1.5 mL 50 mM sodium phosphate buffer (pH 6.0), which were carried out by incubating the reaction at 50 and 75°C (according to the optimum temperature of the enzyme to be determined, data were not shown), respectively, and 180 r/min in a bath shaking incubator. After 6 h, the supernatant of the reaction was collected to analyze the hydrolysate products. XOS were determined by HPLC (Dionex ICS-5000) equipped with a CarboPac PA-200 as the anion-exchange column using 100 mM NaOH and 500 mM NaOAc as the mobile phase at a flow rate of 0.3 mL/min and column temperature of 30°C. Standard four-potential-pulse amperometer was used for detection. The XOS yield and enzymatic efficiency were calculated according to the following equation:

(1) $$\begin{array}{l} \text{XOS yield }(\%)=\text{sum of all XOS }({\text{X}}_{2}-{\text{X}}_{6})/\text{xylan in raw}\\ \text{ material}\times \;100\% \end{array}$$

where, X_2_-X_6_ is the sum of mass concentrations of xylobiose, xylotriose, xylotetraose, xylopentaose, and xylohexaose (mg/mL).

(2) $$\begin{array}{l} \text{Enzymatic hydrolysis rate }(\%)=\text{the mole number of xylose in}\\ \text{ the enzymatic hydrolysate}/\text{the mole number of xylose in the}\\ \text{ raw material }\times \;100\% \end{array}$$

### Optimization of Endo-1,4-β-Xylanase MxynB-8 Hydrolysis of Poplar Sawdust Xylan

The effects of several hydrolysis parameters (temperature, pH, enzyme dosage, and hydrolysis time) on XOS yield from poplar sawdust xylan were determined in batch systems. All reactions were incubated in a shaking water bath using the purified endo-1,4-β-xylanase MxynB-8, setting the poplar sawdust xylan concentration as 2 mg/mL. With the XOS yield as the evaluation index, the effects of different temperature (30, 40, 50, 60, 70, 80, and 90°C), pH (5.0, 5.5, 6.0, 6.5, 7.0, 7.5, and 8.0), enzyme dosage (50, 100, 200, 300, 400, 500, 600, and 800 U/g), and hydrolysis time (3, 6, 9, 12, 15, 18, 21, 24, and 28 h) were optimized. Three groups of parallel tests were set for all conditions. The best MxynB-8 hydrolysis conditions optimized were further used for MxynB-8 and Xln-DT mixture reaction optimization.

### Optimization of Xylose Production From XOS by β-Xylosidase Xln-DT

Endo-1,4-β-xylanase MxynB-8 hydrolysate was used as substrate; further hydrolysis of XOS to produce xylose was carried out by β-xylosidase Xln-DT. For optimization of xylose production, the temperature, pH, and enzyme dosage were optimized. The ranges for the factors were as follows: temperature (30, 40, 50, 60, 70, 80, and 90°C), pH (5.0, 5.5, 6.0, 6.5, 7.0, 7.5, and 8.0), and enzyme dosage (50, 100, 200, 300, 400, and 500 U/g). The xylose concentrations were determined by HPLC (Agilent 1260, USA) equipped with a refractive index detector and Aminex Bio-Rad HPX-87H column using 50 mM H_2_SO_4_ at a flow rate of 0.6 mL/min and column temperature of 55°C.

### Enzymatic Hydrolysis Model of Poplar Sawdust Xylan

Under the optimal conditions, the mode of enzymatic hydrolysis of poplar sawdust xylan by endo-1,4-β-xylanase MxynB-8 and β-xylosidase Xln-DT was studied; the specific design scheme was as follows: (1) only endo-1,4-β-xylanase MxynB-8; (2, 3) simultaneous enzymolysis by MxynB-8 and β-xylosidase Xln-DT; (4) the double-enzyme step hydrolysis: MxynB-8 was added first, and then the suitable temperature was adjusted, and Xln-DT was added; (5) the double-enzyme step hydrolysis: Xln-DT was added first, and then the suitable temperature was adjusted, and MxynB-8 was added; (6) only Xln-DT.

## Results and Discussion

### Expression, Purification, and Characterization of Endo-1,4-β-Xylanase and β-Xylosidase

**Figure 1:**
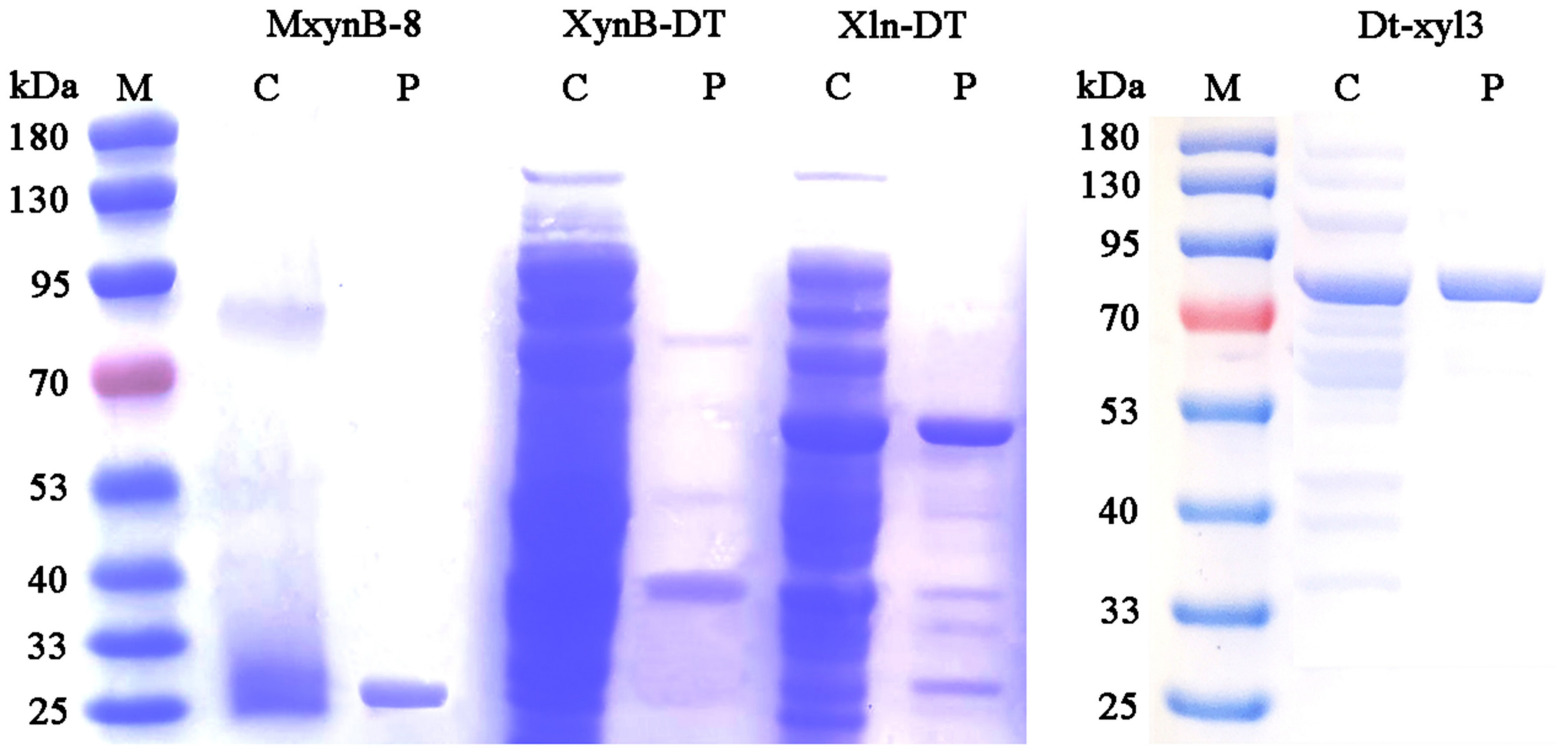
SDS-PAGE analysis of endo-1,4-β-xylanase MxynB-8/XynB-DT and β-xylosidase Xln-DT/Dt-xyl3. M, protein marker; C, crude MxynB-8/XynB-DT/Xln-DT/Dt-xyl3; P, purified protein.

Under the optimal culture conditions, the endo-1,4-β-xylanases MxynB-8 and XynB-DT belonging to GH11 were heterologously expressed in *E. coli* BL21 (DE3) after incubation with 0.1 mM IPTG for 6 h, whereas the β-xylosidase Xln-DT belonging to GH39 and Dt-xyl3 belonging to GH3 were heterologously expressed in *E. coli* BL21 (DE3) after incubation with 0.01 mM IPTG for 14 h. After sonication, almost all the recombinant MxynB-8, XynB-DT, Xln-DT, and Dt-xyl3 were found in the soluble fraction, and the activities of the recombinant endo-1,4-β-xylanase and β-xylosidase were 563.1, 13.7, 5.6, and 5.3 U/mL, respectively. From the initial expression level of enzyme, the MxynB-8 from *A. niger* NL-1 was significantly higher than that of XynB-DT from *D. thermophilum*, ~41.1-fold, which indicated that the industrial application of MxynB-8 was more promising. Then, the soluble fractions were heat-treated in 75°C for 30 min and followed by a Ni^2+^-NTA affinity chromatography. The specific purification steps and yield were shown in Table 1. Finally, the purified endo-1,4-β-xylanase MxynB-8/XynB-DT and β-xylosidase Xln-DT/Dt-xyl3 showed a single band on the SDS-PAGE gel and a molecular mass of ~24, 50, 55, and 80 kDa, respectively, without undesired bands (Figure 1).

**Table 1:** Purification of recombinant protein MxynB-8, XynB-DT, Xln-DT, and Dt-xyl3.

**Enzyme**	**Culture extract**			**Ni affinity chromatography**			**Yield (%)**	**Fold purification**
	**Total activity (U)**	**Total protein (mg)**	**Specific activity (U/mg)**	**Total activity (U)**	**Total protein (mg)**	**Specific activity (U/mg)**		
MxynB-8	56,312.8	97.7	576.38	43,923.9	48.2	910.68	78.1	1.58
XynB-DT	13,766.1	2,935.2	4.69	9,553.7	352.2	27.12	69.4	5.78
Xln-DT	560.4	190.5	2.94	318.3	37.2	8.56	56.8	2.91
Dt-xyl3	532.4	287.9	1.85	285.4	46.3	6.16	53.4	3.35

*Substrate for MxynB-8 and XynB-DT was beechwood xylan, while substrate for Xln-DT and Dt-xyl3 was p-nitrophenyl-β-d-xylopyranoside*.

### Enzymatic Specificity of MxynB-8 and XynB-DT to the Substrate

The endo-1,4-β-xylanase MxynB-8 and XynB-DT were tested for the specific activities to the different substrates and showed preference for xylan from birchwood, followed by beechwood xylan, poplar sawdust xylan, and oat spelt xylan (Table 2). Compared with the xylan from Gramineae (oat spelt xylan), these two endo-1,4-β-xylanases were more suitable for the degradation of xylan from broadleaf plants (68.4, 65.5, and 63.8% XOS yields for birchwood, beechwood, and poplar sawdust xylan, respectively, by MxynB-8, whereas 51.9, 45.9, and 54.4% XOS yields for birchwood, beechwood, and poplar sawdust xylan, respectively, by XynB-DT). Moreover, MxynB-8 and XynB-DT showed very low affinities for carboxymethylcellulose (CMC), which suggests that these two endo-1,4-β-xylanases do not have cellulose degradation function, as is the case of endo-1,4-β-xylanase of *Streptomyces* sp. (Georis et al., 2000) and *Caulobacter crescentus* (Jacomini et al., 2020). Cellulose-free endo-1,4-β-xylanase can remove hemicellulose compounds selectively with minimal cellulose loss, which is more profitable industrial processes, such as pulp bleaching, textile biorefining treatment, and food industry (Belfaquih et al., 2002).

**Table 2:** Enzymatic hydrolysis of different xylans and other substrates in 6 h by MxynB-8 and XynB-DT.

**Source**	**MxynB-8**							**XynB-DT**						
	**XOS yield (%)**	**Distribution of X1–X6 (%)**						**XOS yield (%)**	**Distribution of X1–X6 (%)**					
		**X1**	**X2**	**X3**	**X4**	**X5**	**X6**		**X1**	**X2**	**X3**	**X4**	**X5**	**X6**
Birchwood xylan	68.4	10.2	41.1	2.9	—	2.3	43.4	51.9	14.0	68.9	14.6	—	—	2.5
Beechwood xylan	65.5	9.6	37.8	3.0	—	2.4	47.2	45.9	15.8	67.5	2.9	10.3	3.4	—
Oat spelt xylan	42.6	19.1	62.6	14.7	—	3.5	—	32.8	17.2	72.9	4.1	—	—	5.8
Poplar sawdust xylan	63.8	5.2	36.4	6.0	6.9	2.2	43.2	54.4	1.8	46.0	11.3	8.6	2.8	29.5
CMC	ND							ND						

*X1, xylose; X2, xylobiose; X3. xylotriose; X4. xylotetraose; X5. xylopentaose; X6, xylohexaose; XOS, xylooligosaccharides; CMC, carboxymethylcellulose; ND, not detected*.

**Figure 2:**
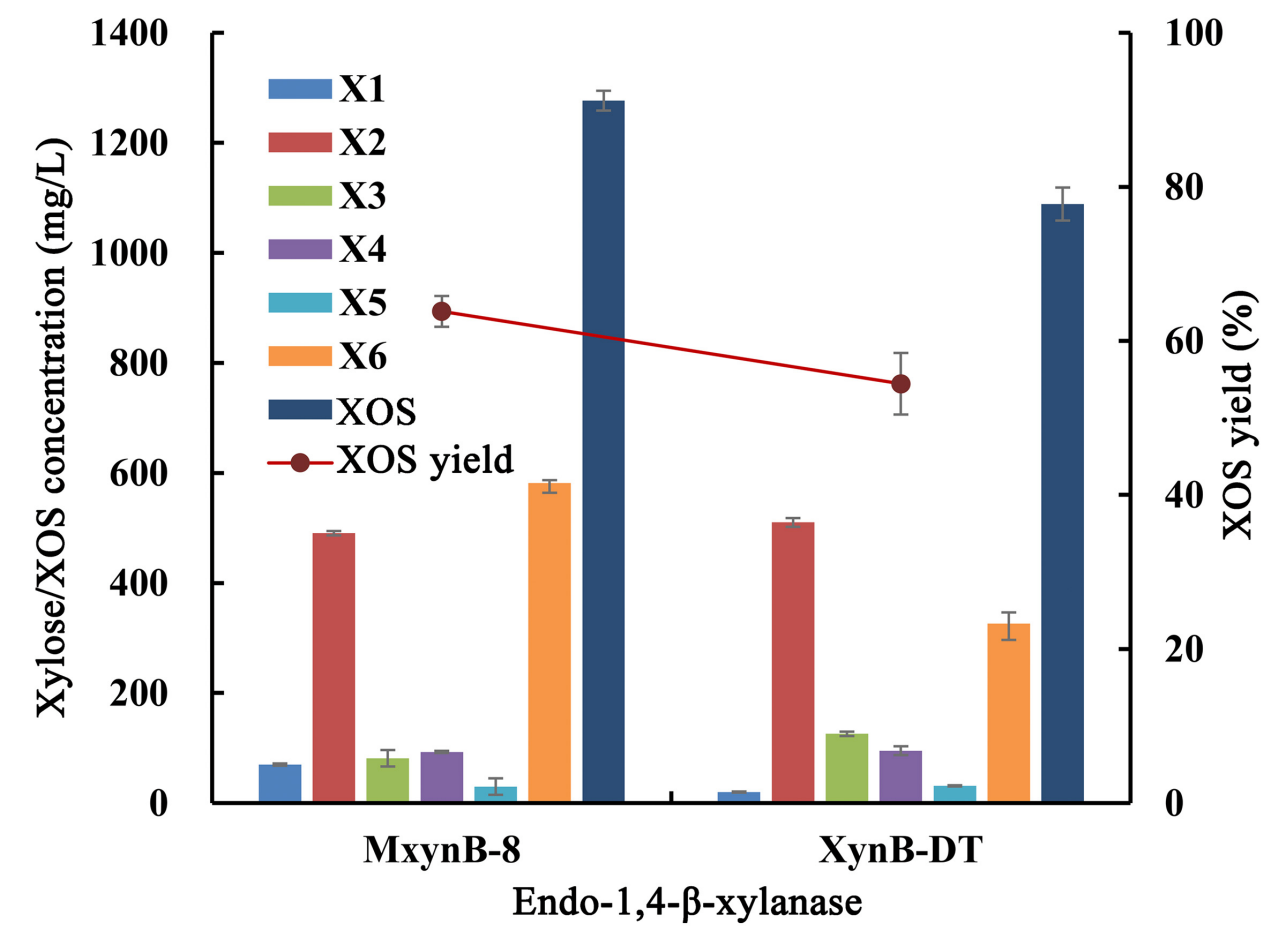
Degradation products and XOS yield of poplar sawdust xylan by endo-1,4-β-xylanase MxynB-8/XynB-DT. X1, xylose; X2, xylobiose; X3, xylotriose; X4, xylotetraose; X5, xylopentaose; X6, xylohexaose; XOS, xylooligosaccharides.

In consideration of beechwood and birchwood, xylans are commercially difficult to obtain by most suppliers, or else the cost of the available products is very high. In addition, the composition and structure of these substrates derived from broadleaf are similar, so the performance of the endo-1,4-β-xylanases against these compounds is also equivalent. In the later experiments, poplar sawdust xylan was used as substrate to investigate the hydrolysis efficiency of these two endo-1,4-β-xylanases. After 6-h hydrolysis, MxynB-8 and XynB-DT both could degrade the poplar sawdust xylan into XOS, with the XOS yield of 63.8 and 54.4%, respectively (Figure 2). Xylobiose and xylohexaose are the main components in the hydrolysate, with a small amount of xylose, xylotriose, xylotetraose, and xylopentaose. Similar results were obtained for endo-1,4-β-xylanases from *Streptomyces* sp. (Kholis et al., 2015) and *Massilia* sp. (Xu et al., 2016), which degrade beechwood and wheat xylan specifically, and showed no detectable enzyme activity to other polysaccharides with different compositions. As the endo-1,4-β-xylanase MxynB-8 from *A. niger* has excellent expression capacity, its protein expression is significantly higher than that of XynB-DT from *D. thermophilum*, which is 41.1-fold of its expression. Considering the cost of enzyme, the application of endo-1,4-β-xylanase MxynB-8 from *A. niger* in hemicellulose degradation is more suitable.

### Substrate Specificity and Effect of Sugar on Xln-DT and Dt-xyl3 Activity

In addition to xylosyl recognition ability, β-xylosidase also has different catalytic ability to other types of glycosyl, such as glucosyl, arabinopyranosyl, galactosyl, etc. Shi et al. (2013) cloned a GH3 β-xylosidase from *Thermotoga thermarum*, which could hydrolyze not only xylose but also arabinose. Zhang et al. (2019b) reported a GH3 β-xylosidase from *T. petrophila* could hydrolyze not only xylosyl, but also glucose and arabinopyranosyl groups. In order to further analyze the substrate specificity and kinetic constant (*K*_m_ and *V*_max_) of two β-xylosidases, the enzyme activities of the purified enzymes Xln-DT and Dt-xyl3 were detected, respectively, by using *p*NPX, *p*NPG, *o*NPG, *p*NPGal, *p*NPR, *p*NPA, cellobiose, xylobiose, and CMC. As shown in Table 3, both the enzyme Xln-DT and Dt-xyl3 were able to hydrolyze *p*NPX and xylobiose with excellent efficiency and had few ability to hydrolyze *p*NPG and *p*NPA, whereas no activity was detected upon *o*NPG, *p*NPGal, *p*NPR, and CMC. *p*NPG was hydrolyzed at 25.9 and 21.6% of that of *p*NPX by Xln-DT and Dt-xyl3, respectively, whereas for *p*NPA the relative activities were 44.4 and 30.8%, respectively. The dependence of the rate of the enzymatic reaction on the substrates concentration followed Michaelis–Menten kinetics, with *K*_m_ and *V*_max_ values of 1.66 mM and 13.58 U/mg for Xln-DT, respectively, and 0.83 mM and 19.05 U/mg for Dt-xyl3, respectively, using the *p*NPX as the substrate. For xylobiose, the *K*_m_ and *V*_max_ values were 0.93 mM and 714.29 U/mg for Xln-DT, respectively, which were higher than that of Dt-xyl3. Thus, the degradation efficiency of XOS by Xln-DT is higher than that by Dt-xyl3.

**Table 3:** Kinetic parameters and specific activities of recombinant Xln-DT and Dt-xyl3.

**Substrate**	**Xln-DT**			**Dt-xyl3**		
	***K*_**m**_ (mM)**	***V*_**max**_ (U/mg)**	**Relative activity (%)**	***K*_**m**_ (mM)**	***V*_**max**_ (U/mg)**	**Relative activity (%)**
*p*NPX	1.66	13.58	100.0	0.83	19.05	100.0
*p*NPG	3.67	6.03	25.9	2.39	10.90	21.6
*o*NPG	ND			ND		
*p*NPGal	ND			ND		
*p*NPR	ND			ND		
*p*NPA	1.02	3.89	44.4	2.04	28.68	30.8
Cellobiose	11.00	217.39	—	2.28	277.78	—
Xylobiose	0.93	714.29	—	4.18	666.7	—
CMC	ND			ND		

*pNPX, p-nitrophenyl-β-d-xylopyranoside; pNPG, pNP-β-d-glucoside; oNPG, oNP-β-d-glucoside; pNPGal, p-nitrophenyl-β-d-galactopyranoside; pNPR, p-nitrophenyl-α-l-rhamnopyranoside; pNPA, pNP-α-l-arabinofuranoside; CMC, carboxymethylcellulose; ND, not detected*.

*Values shown are the mean of duplicate experiments, and the variation about the mean is below 5%*.

**Figure 3:**
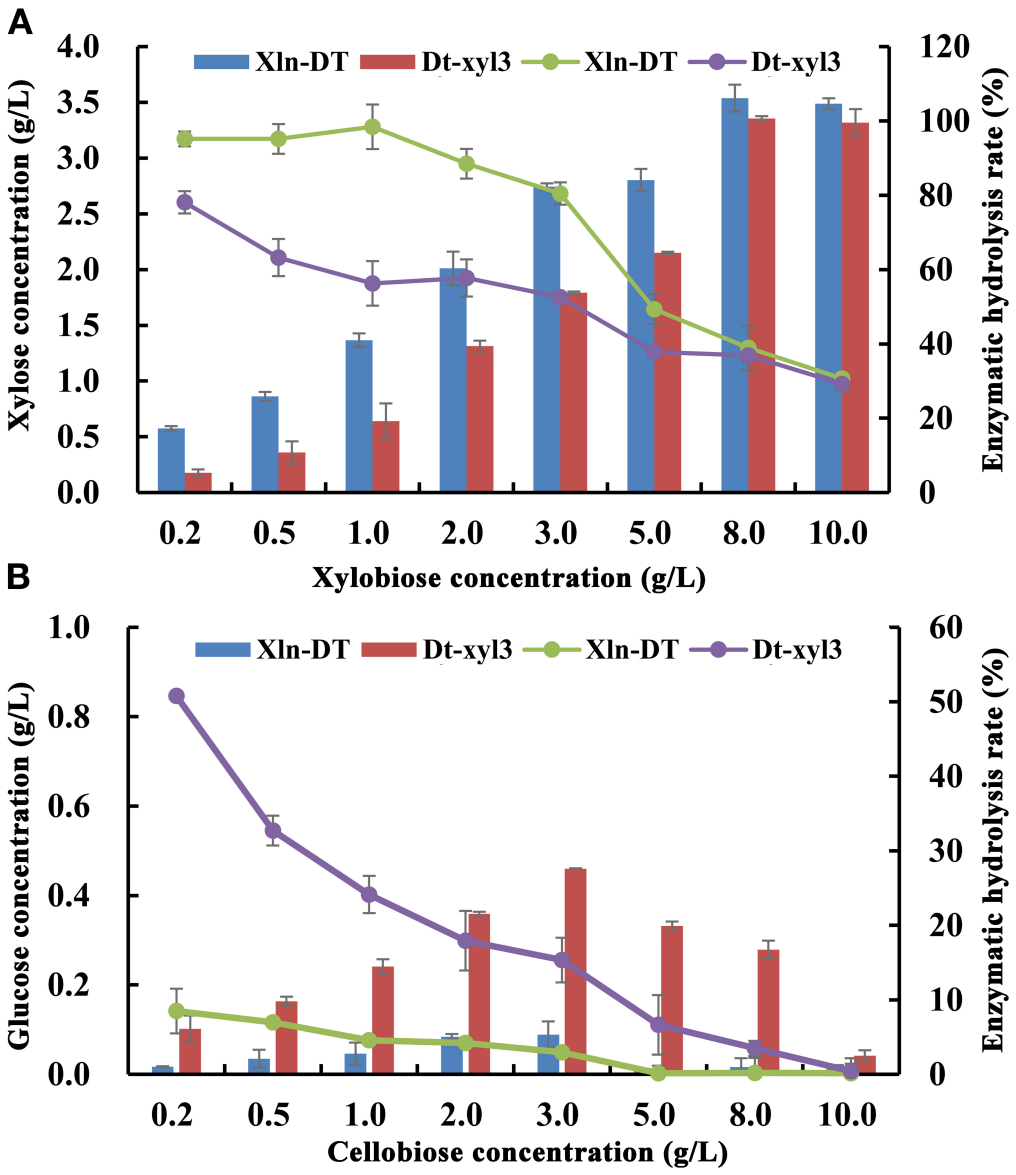
Analysis of xylobiose and cellobiose hydrolyzed by recombinant β-xylosidase Xln-DT and Dt-xyl3 [**(A)** Xylobiose; **(B)** Cellobiose; column: xylose/glucose concentration; line: enzymatic hydrolysis rate].

Production of xylose and glucose from different concentrations of xylobiose and cellobiose by the purified Xln-DT and Dt-xyl3 were determined. With the increase of the xylobiose concentration, xylose was also accumulated (Figure 3). When the concentration of xylobiose was below 3 g/L, the yield of Xln-DT enzymatic hydrolysis xylose could be maintained 80.5%, whereas the yield of Dt-xyl3 enzymatic hydrolysis xylose could be only maintained 52.6%. In addition, the hydrolysis ability of cellobiose by Xln-DT was significantly lower than that by Dt-xyl3. When 0.2 g/L of cellobiose was used as substrate, the enzymatic hydrolysis yield of Dt-xyl3 was 50.7%, whereas that of Xln-DT was 8.5%. Therefore, compared with Dt-xyl3, Xln-DT is more suitable for hemicellulose hydrolysis, which only hydrolyzed β-1,4-xyloside bond, but the hydrolysis rate of β-1,6-glucoside bond is very low, thus can reduce the hydrolysis loss of cellulose.

**Figure 4:**
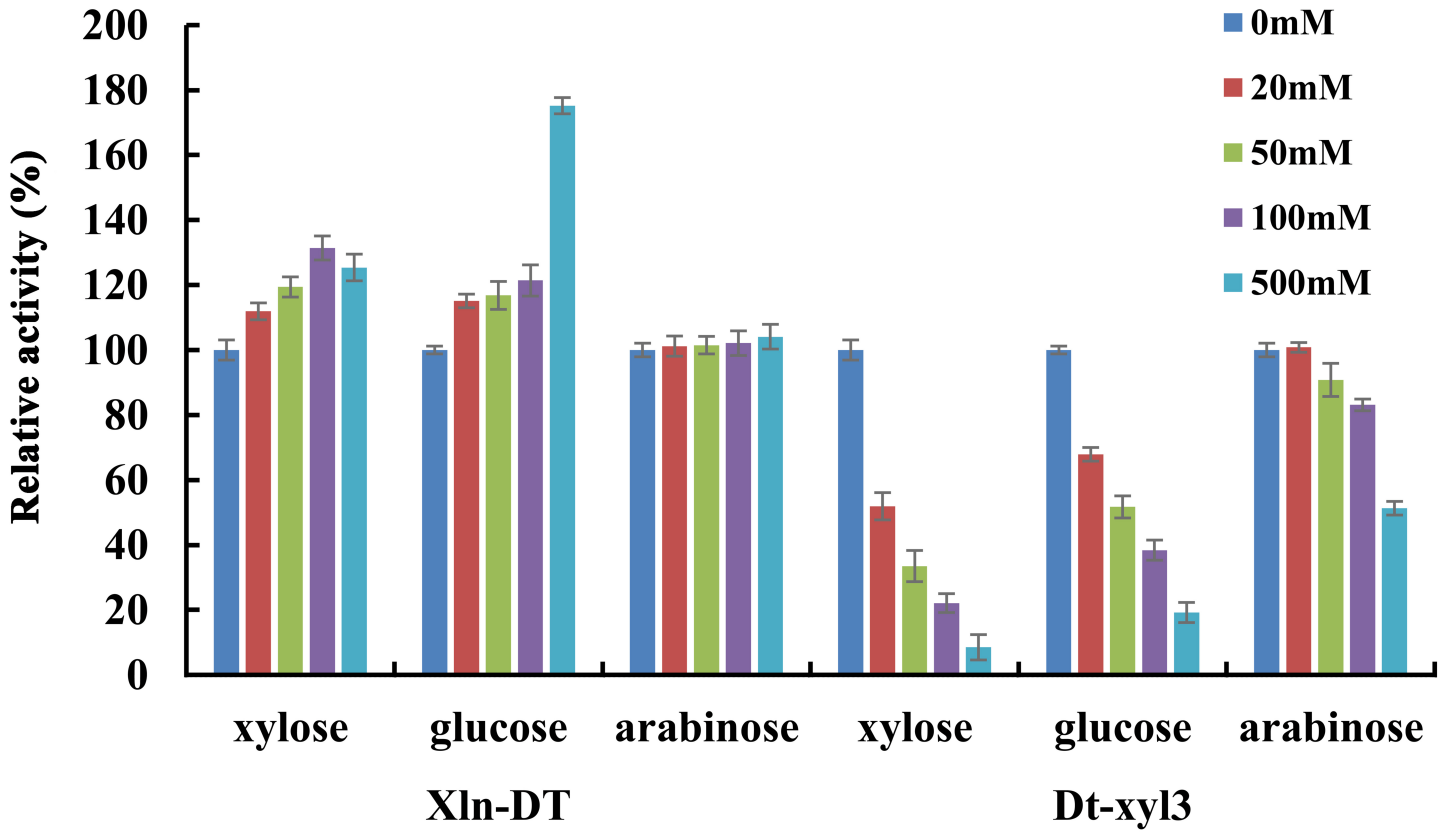
The effects of xylose/glucose/arabinose on the activities of recombinant β-xylosidase Xln-DT and Dt-xyl3 (column: 0, 20, 50, 100, or 500 mM concentration of sugar). The activity of β-xylosidase enzyme without xylose/glucose/arabinose was defined as relative 100%. Influence of xylose/glucose/arabinose on enzyme activity with *p-*nitrophenyl-β-d-xylopyranoside.

Glycoside hydrolases, such as β-glucosidase, β-xylosidase, and α-rhamnosidase, are sensitive to sugars. Only a few of the reported glycoside hydrolases are sugar tolerant and stimulated (Liu et al., 2017; Zhang et al., 2019a). In previous studies, β-xylosidases with sugar tolerance and stimulation can improve the efficiency of hemicellulose degradation, which are more suitable for industrial application. In the process of hemicellulose hydrolysis, xylose, glucose, and arabinose are the main monosaccharides, high concentrations of which would inhibit β-xylosidase activity. Therefore, we investigated the inhibitory effects of xylose, glucose, and arabinose on the activities of two β-xylosidases Xln-DT and Dt-xyl3 at different concentrations (Figure 4). As shown in Figure 4, the GH39 β-xylosidases Xln-DT had excellent glucose and xylose tolerance, which the relative enzyme activity of Xln-DT increased by 25.4 and 75.2%, respectively, in the presence of xylose and glucose of 500 mM. And the arabinose had no inhibitory effect within 500 mM to Xln-DT. However, the three monosaccharides had obvious inhibitory effect on the GH3 β-xylosidases Dt-xyl3. Among them, xylose had the most obvious inhibitory effect on Dt-xyl3. When the xylose concentration was 20 mM, the relative activity of Dt-xyl3 was only 51.7%, whereas when the xylose concentration was 500 mM, Dt-xyl3 was completely inhibited. Then there was the inhibition of glucose on Dt-xyl3, which the relative enzyme activity of Dt-xyl3 was reduced to 51.7 and 19.2% in 50 and 500 mM of glucose, respectively. Arabinose had the least inhibitory effect, and its residual enzyme activity of Dt-xyl3 reached 51.3% at 500 mM. These results were also close to the reported literature, which shows the β-xylosidases from GH3 family had very poor tolerance to xylose or glucose (Zhang et al., 2019b). All the results above suggested that Xln-DT was more beneficial and suitable to hemicellulose degradation or other industrial applications without the product feedback inhibition than Dt-xyl3.

### Optimization of Hydrolysis Conditions of Poplar Sawdust Xylan by MxynB-8

Xylan, as the second most plentiful polysaccharide in plant cell walls, accounts for 20–30% of the secondary cell wall of the dicots. With the processing and utilization of poplar wood resources, a large number of wood residues, sawdust, and other residues are produced with a global yield 0.8 tons per m^3^ of poplar wood (Yan et al., 2015). Also, the poplar sawdust is a by-product of poplar wood with a high hemicellulose content of 20–35%, of which xylosyl accounts for 30–40% of the total sugar. Poplar sawdust used in this study contained 23.93% of xylan. Poplar sawdust xylan was extracted by alkali method; 17.97 ± 0.12 g alkaline extractive with a xylan content of 88.69 ± 0.52% was obtained from 100 g dried poplar sawdust, resulting in an extraction rate of 66.61 ± 0.28%.

**Figure 5:**
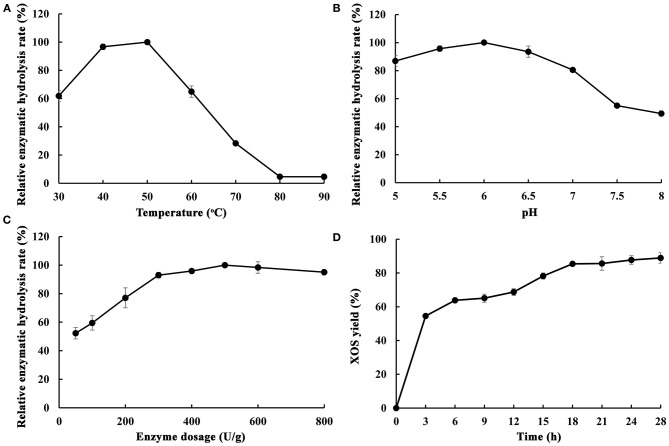
Optimization of different enzymatic hydrolysis conditions of poplar sawdust xylan by recombinant endo-1,4-β-xylanase MxynB-8 [**(A)** The effect of temperature on the enzymatic hydrolysis rate; **(B)** The effect of pH on the enzymatic hydrolysis rate; **(C)** The effect of enzyme dosage on the enzymatic hydrolysis rate; **(D)** The effect of time on the enzymatic hydrolysis rate]. The maximum hydrolysis rate measured at a certain temperature/pH/enzyme dosage was defined as relative 100%.

As is well-known, endo-1,4-β-xylanase is a key enzyme for xylan hydrolysis and eventually for XOS production. The composition of endo-1,4-β-xylanases from different microorganisms are different, which lead to the difference of xylan hydrolysis ability. In order to understand the effect and suitable conditions of enzymatic hydrolysis of poplar sawdust xylan by MxynB-8, the effects of key parameters, such as temperature, pH, enzyme dosage, and hydrolysis time on enzymatic hydrolysis efficiency were studied, and the products after enzymatic hydrolysis were analyzed (Figure 5).

The effect of temperature on the enzymatic hydrolysis of poplar sawdust xylan was obvious. In a certain range (30–50°C), with the increase of temperature, it could accelerate the conversion rate of enzyme substrate intermediate into products, while the high temperature would affect the stability of the enzyme and reduce the enzymatic hydrolysis rate. As shown in Figure 5A, the optimum temperature for enzymatic hydrolysis of poplar sawdust xylan by MxynB-8 was 50°C. It is quite different from the reported optimal temperature of endo-1,4-β-xylanase XynB-DT from *D. thermophilum* (Tong et al., 2020) for enzymatic hydrolysis of poplar sawdust xylan, which indicated that temperature has great influence on the enzymatic hydrolysis efficiency, and such temperature difference is related to the source and structure of the enzyme. Considering the cost of the enzyme used in the industrial application, the temperature of the degradation should be minimized.

To investigate the optimal pH of MxynB-8, at the optimum temperature of 50°C, the substrate concentration of poplar sawdust xylan was set to 2 mg/mL and hydrolyzed for 12 h in different pH values, and the maximum enzymatic yield is defined as relative 100%. The optimal pH of enzymatic reaction is shown in Figure 5B. With the increase of pH values, the enzymatic hydrolysis of xylan was first increased (pH 5–6) and then decreased (pH 6–8). When the pH was 6.0, the hydrolysis efficiency of poplar sawdust xylan by MxynB-8 was the highest, which was also consistent with the optimal pH of the enzyme in beechwood degradation. Moreover, when the pH value is too low, the self-hydrolysis effect of xylan is enhanced, and if the pH value is too high, the activity of endo-1,4-β-xylanase MxynB-8 will be affected. Considering the above results, pH 6.0 was selected as the optimal pH for poplar sawdust xylan degradation.

The enzyme dosage has a great influence on the enzymatic hydrolysis efficiency. If the enzyme dosage is too low, it is easy to cause insufficient enzymatic hydrolysis, and if the enzyme dosage is too large, the cost will increase. Therefore, it is very important to select the appropriate amount of enzyme. Setting the concentration poplar sawdust xylan as 2 mg/mL and the enzymolysis temperature and pH at 50°C and 6.0, respectively, the optimal enzyme dosage of MxynB-8 is shown in Figure 5C. Under the given conditions, with the increase of enzyme dosage of MxynB-8, the hydrolysis efficiency gradually increased. When the enzyme dosage of MxynB-8 exceeded 500 U/g, the enzymatic hydrolysis efficiency remained unchanged. The reason may be that the number of binding sites between xylan and MxynB-8 is limited. At the beginning of enzymatic reaction, with the increase of MxynB-8 amount, the long chain of poplar sawdust xylan was rapidly hydrolyzed, resulting in a large number of XOS. However, when all these binding sites are occupied by MxynB-8, further increase of enzyme dosage will only increase the invalid adsorption of enzyme and substrate. As a result, the enzymatic hydrolysis of MxynB-8 was inhibited. Therefore, the optimal enzyme dosage of MxynB-8 was 500 U/g.

Endo-1,4-β-xylanase preferentially acts on long-chain xylan to produce XOS and then hydrolyzes the XOS into mainly xylobiose and xylotriose and finally to monosaccharide by adding β-xylosidase. If the hydrolysis time is too short, the enzymolysis is not sufficient. Because of the limitation of the temperature stability of the enzyme, excessive extension of the enzymatic hydrolysis time is not conducive to the production of appropriate XOS. Therefore, in order to obtain as many XOS as possible, the enzymolysis time must be optimized. Samples were taken after 3, 6, 9, 12, 15, 18, 21, 24, and 28 h at the optimum temperature, pH, and enzyme dosage; the results are shown in Figure 5D. When the enzymolysis time was <18 h, the enzymolysis efficiency of MxynB-8 increased rapidly with the increase of time. In 3–12 h enzymatic hydrolysis time, xylobiose, xylotriose, xylopentose, and xylohexaose were the main XOS detected. In 12–18 h, the amount of xylobiose and that of xylotriose continued to increase, whereas the content of xylohexaose decreased slowly, which indicates that endo-1,4-β-xylanase hydrolyzed xylan into XOS continuously. The results confirmed that hydrolysis pattern of MxynB-8 was due to cleaving of the inner β-1,4-xylocoside bonds randomly, which was as similar as the endoxylanases from *Streptomyces ipomoeae* (Xian et al., 2019) and *Trichoderma reesei* (Oliveira et al., 2018). When the enzymolysis time was more than 18 h, the growth rate of enzymatic hydrolysis efficiency obviously slowed down and even had a downward trend. This is because the enzyme activity of endo-1,4-β-xylanase MxynB-8 decreased after fully combining with the substrate, and with the extension of time, some XOS would also be self-hydrolyzed, resulting in the break of β-1,4-xyloside chain. Therefore, the optimal enzymolysis time of MxynB-8 was 18 h. After optimization, XOS yield of MxynB-8 was 85.5%, which was higher than the values (between 72.5 and 73.9%) reported by acid hydrolysis (Huang et al., 2019). Among the hydrolysates, xylobiose and xylotriose were the main hydrolysates, accounting for 75.5% (with a concentration of 1.29 g/L in hydrolysate) and 24.3% (with a concentration of 0.42 g/L in hydrolysate) of the total hydrolysates, respectively, and contained a small amount of xylose and xylohexaose. The ratio of xylobiose in the xylose-based sugars released by MxynB-8 was higher than the previously reported 19.56 and 51.6% (Azelee et al., 2016; Sepulchro et al., 2020) and lower than reported 85.99% (Xian et al., 2019). In addition, the enzymatic efficiency based on the poplar sawdust xylan in the raw material was 30.5%, which is slightly higher than or similar to other reported enzymatic hydrolysis results (Azelee et al., 2016; Jacomini et al., 2020; Sepulchro et al., 2020). These results indicate that MxynB-8 has a promising biotechnological potential to transform poplar sawdust xylan into XOS, which can contribute to valorization of underutilized agricultural and forestry wastes.

### Optimization of Hydrolysis Conditions of XOS From Poplar Sawdust Xylan by Xln-DT

**Figure 6:**
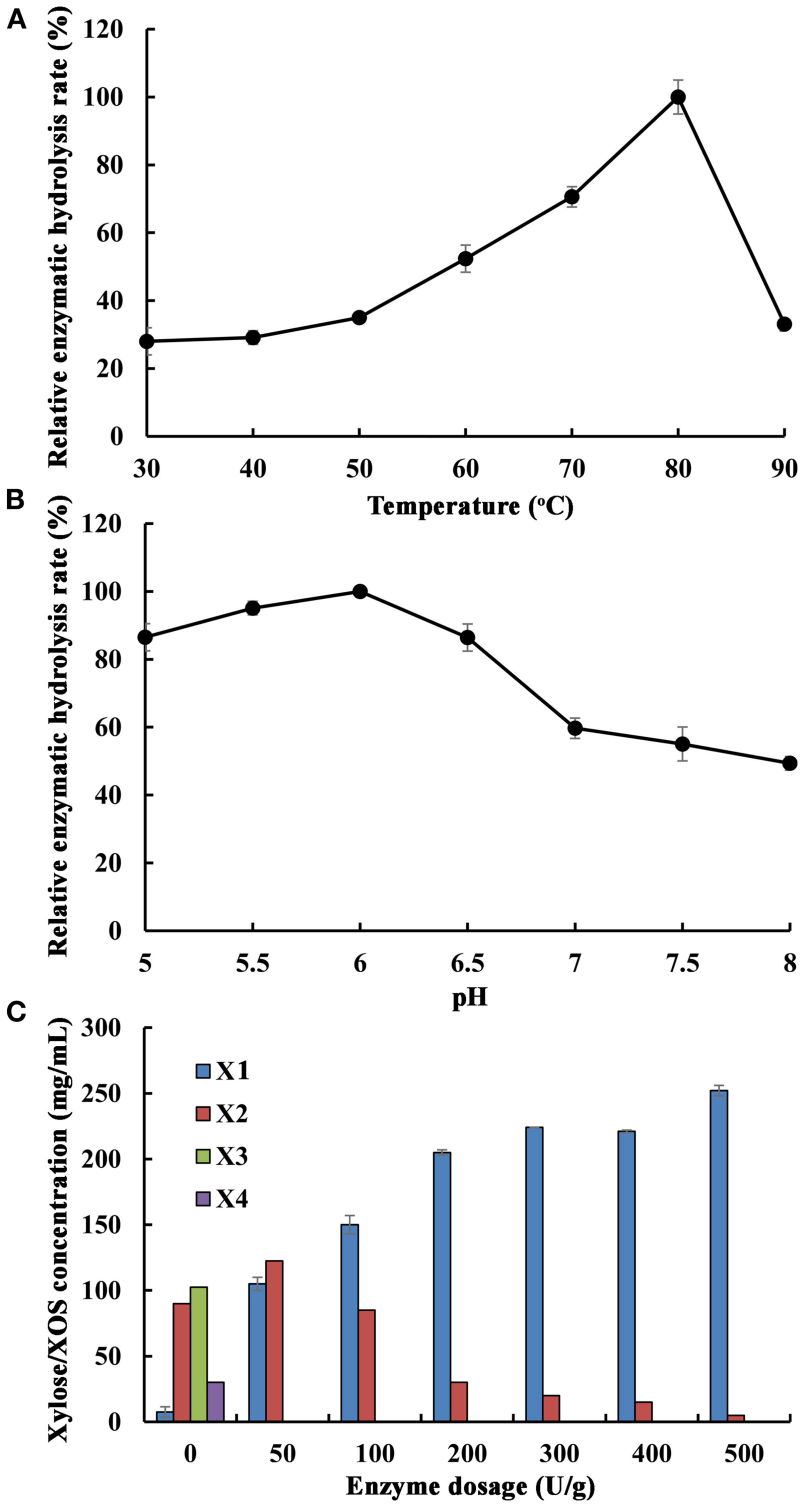
Optimization of different enzymatic hydrolysis conditions of xylooligosaccharides from poplar sawdust xylan by recombinant β-xylosidase Xln-DT (**(A)** The effect of temperature on the enzymatic hydrolysis rate; **(B)** The effect of pH on the enzymatic hydrolysis rate; **(C)** The effect of enzyme dosage on the enzymatic hydrolysis rate). X1, xylose; X2, xylobiose; X3, xylotriose; X4, xylotetraose. The maximum hydrolysis rate measured at a certain temperature and pH was defined as relative 100%.

Generally, β-1,4-xylosidase can mainly degrade XOS from the non-reducing end to produce xylose. In fermentation processes, using xylose as a carbon source to produce ethanol and xylitol is of great industrial relevance (Sderling and Pienihkkinen, 2020). With deciding the optimal hydrolysis conditions of XOS from poplar sawdust xylan by Xln-DT, the effects of temperature, pH, and enzyme dosage were studied in the second stage treatment. The main hydrolysis product, xylose, the product of the reaction catalyzed by XOS, is similar to that previously reported. After extended incubation times, only a small amount of xylose was continued to release, which rendered the optimization of the hydrolysis time less necessary. The purified β-1,4-xylosidase Xln-DT showed a concentration of 100 U/g was added into the poplar sawdust xylan hydrolyzed with the concentration of 1.6 g/L. By using the xylose content in the hydrolysate as the detection index, the optimal temperature and pH of Xln-DT in degrading XOS are shown in Figures 6A,B. After 4-h hydrolysis time, the xylose concentration was increased within a temperature range of 30–80°C, optimal temperature of which was 80°C, and the optimal pH was 6.0. We set the temperature at 80°C and pH 6.0; the effect of Xln-DT dosage on the enzymatic hydrolysis efficiency was optimized (Figure 6C). The results showed that XOS (xylobiose, xylotriose, etc.) could be continuously hydrolyzed into d-xylose by adding a small amount of Xln-DT (0–100 U/g). When the Xln-DT dosage was 500 U/g, the xylobiose and xylotriose were hydrolyzed to d-xylose wholly, with the enzymatic hydrolysis rate reaching the maximum 32.2%, and d-xylose was the main component in the end-products. After that, the enzymolysis efficiency was basically unchanged by adding more Xln-DT, so the Xln-DT dosage was set as 500 U/g.

### Synergistic Enzymatic Hydrolysis of Poplar Sawdust Xylan by MxynB-8 and Xln-DT

**Figure 7:**
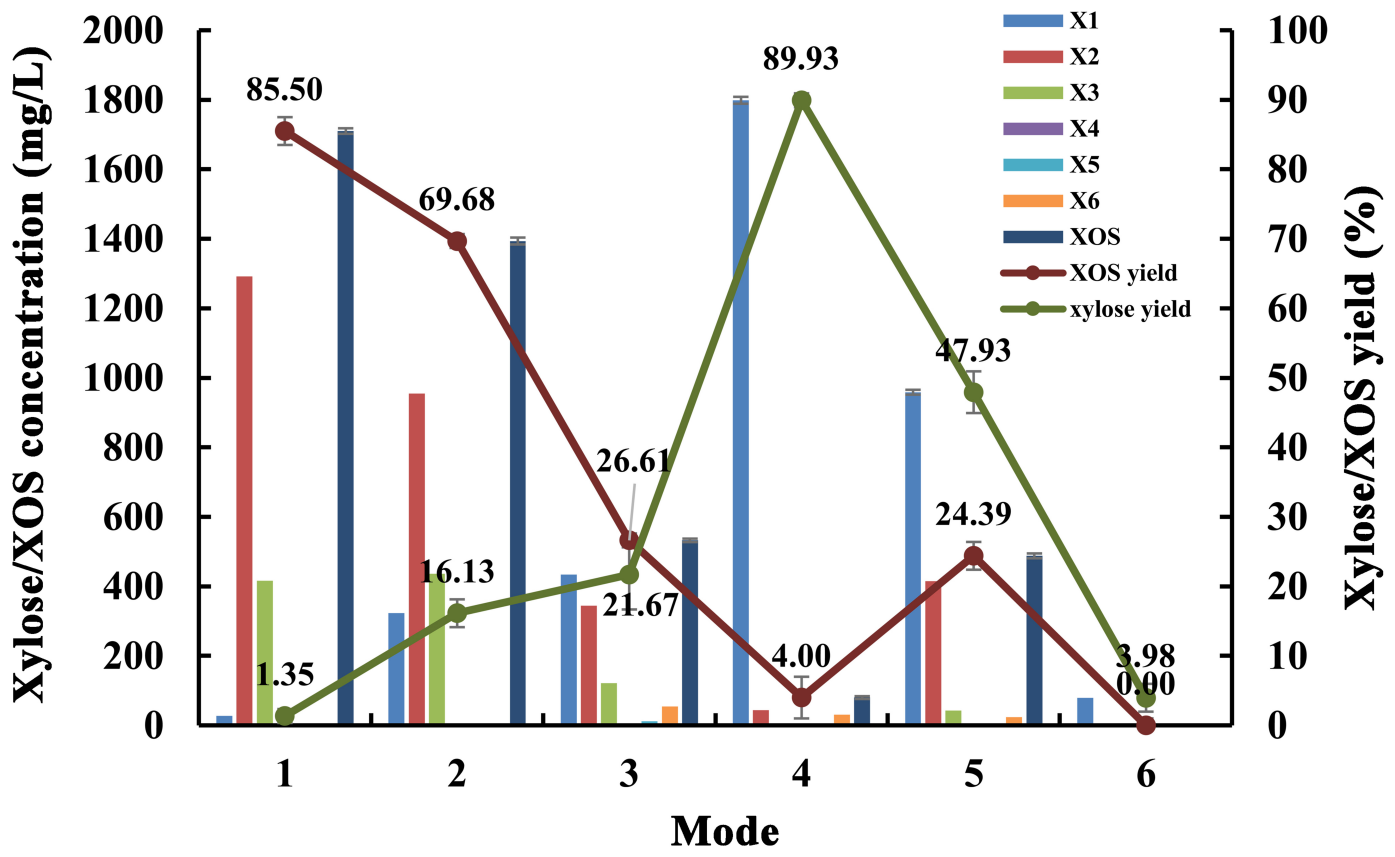
Effects of different enzymatic hydrolysis modes on degradation products and yield of poplar sawdust xylan (Mode 1: only MxynB-8 at 50°C and pH 6.0 for 18 h; Mode 2: MxynB-8 and Xln-DT were added simultaneously at 50°C and pH 6.0 for 18 h; Mode 3: MxynB-8 and Xln-DT were added simultaneously at 80°C and pH 6.0 for 18 h; Mode 4: first add MxynB-8 at 50°C and pH 6.0 for 18 h and then Xln-DT were added at 80°C and pH 6.0 for 4 h; column: xylose/XOS concentration; line: xylose/XOS yield). X1, xylose; X2, xylobiose; X3, xylotriose; X4, xylotetraose; X5, xylopentaose; X6, xylohexaose; XOS, xylooligosaccharides.

In addition, we used endo-1,4-β-xylanase MxynB-8 and β-1,4-xylosidase Xln-DT to synergetic degrade, the enzymolysis efficiency of which was evaluated by different enzymolysis modes. The degree of synergy was calculated as a means of assessing the synergistic activities in the context of poplar sawdust xylan degradation. Synergy of endo-1,4-β-xylanase and β-xylosidase against xylan could benefit for the enzymatic hydrolysis of xylan to xylose and further reduce cost. The synergistic effects between endo-1,4-β-xylanase MxynB-8 and β-xylosidase Xln-DT on poplar sawdust xylan degradation were determined by simultaneous or sequential addition, which is shown in Table 4. The yield of XOS and xylose from the degradation products under different enzymatic hydrolysis modes is shown in Figure 7. When adding MxynB-8 alone, XOS, mainly xylobiose, xylotriose, and xylohexaose, were the main hydrolysates of poplar sawdust xylan, with the XOS yield 85.5%, and low amounts of xylose were released. When employing MxynB-8 together with Xln-DT (Modes 2 and 3), the xylose yield of poplar sawdust xylan was increased by 91.6 and 93.8% for Modes 2 and 3, respectively. However, in Mode 3, the XOS yield (26.61%) was lower than those of Modes 1 (85.50%) and 2 (69.68%). The reason may be that MxynB-8 is a mesophilic enzyme; under high temperature, such as 80°C, the enzyme was easily inactivated, which leads to the decrease of enzymatic hydrolysis efficiency. At the double-enzyme step hydrolysis, first addition of MxynB-8 and then of Xln-DT at the suitable temperature, the xylose yield of poplar sawdust xylan was the highest (89.93%), which showed synergism effects between MxynB-8 and Xln-DT during hydrolysis, in which xylan was degraded to XOS by the endo-1,4-β-xylanase MxynB-8 and then effectively cleft to xylose by the β-xylosidase Xln-DT. The degree of synergy was calculated and showed that it was 15.89 in Mode 4, which was higher than that of Xyl43A from *H. insolens* (Yang X. et al., 2015) and Ac-Abf51A from *Alicyclobacillus* sp. (Yang W. X. et al., 2015). These results further underscore the mutual synergistic activity of endo-1,4-β-xylanase and β-xylosidase. At Mode 5, at which Xln-DT was added first, suitable temperature was adjusted, and MxynB-8 was added. The release of xylose (958.58 mg/L) was lower than that of Mode 4 (1,798.57 mg/L), which showed that xylan with a high degree of polymerization could not be affected by the first addition of Xln-DT. When endo-1,4-β-xylanase MxynB-8 was added, MxynB-8 hydrolyzed xylan macromolecules into XOS, and then Xln-DT could perform its function. This situation was also verified in Mode 6. When only Xln-DT was added, the yield of xylose from poplar sawdust xylan was very low, only 4.0%. To sum up, when only endo-1,4-β-xylanase MxynB-8 was added, XOS, such as xylobiose and xylotriose, were the main hydrolysates. In addition, xylose was the main component in the end-product after β-xylosidase Xln-DT was added. The greatest synergy degree (7.8-fold) was found in the simultaneous enzyme additions of MxynB-8 and Xln-DT, which suggested a pronounced synergistic effect of GH39 Xln-DT with the GH11 endo-1,4-β-xylanase on poplar sawdust xylan degradation. Moreover, as the optimal pH of MxynB-8 and Xln-DT was both 6.0, there was no need to change the pH value in the two-step hydrolysis system and adjusted only to the optimal temperature by each, which greatly reduced the cost of enzymatic hydrolysis. It is also suggested that MxynB-8 and Xln-DT have a good synergistic effect in bioconversion of xylan-rich lignocellulosic materials, such as poplar sawdust to produce XOS and xylose and further reduce cost.

**Table 4:** Simultaneous or sequential hydrolysis reactions by endo-1,4-β-xylanase MxynB-8 and β-xylosidase Xln-DT against poplar sawdust xylan substrate.

**Enzyme added**				**Poplar sawdust xylan**	
**First reaction**	**Reaction conditions**	**Second reaction**	**Reaction conditions**	**Xylose concentration (μmol)**	**Synergy**
MxynB-8	50°C, pH 6.0, dosage of 500 U/g, 18 h	None	—	26.94	—
MxynB-8+Xln-DT	50°C, pH 6.0, dosage of 500 and 100 U/g, 18 h	None	—	322.54	2.03
MxynB-8+Xln-DT	80°C, pH 6.0, dosage of 500 and 100 U/g, 18 h	None	—	433.48	3.07
MxynB-8	50°C, pH 6.0, dosage of 500 U/g, 18 h	Xln-DT	80°C, pH 6.0, dosage of 100 U/g, 4 h	1,798.57	15.89
Xln-DT	80°C, pH 6.0, dosage of 100 U/g, 4 h	MxynB-8	50°C, pH 6.0, dosage of 500 U/g, 18 h	958.58	8.01
Xln-DT	80°C, pH 6.0, dosage of 100 U/g, 4 h	None	—	79.50	—

*Simultaneous or sequential reactions refer to the reactions with two enzymes added simultaneously or sequentially, respectively*.

*Synergy degree is defined as the ratio of xylose equivalents from simultaneous or sequential enzyme combinations to the sum of that released by the individual enzymes*.

## Conclusion

In this study, a GH11 endo-1,4-β-xylanase MxynB-8 and a GH39 β-xylosidase Xln-DT were used in degradation of poplar sawdust xylan. Compared with other characterized endo-1,4-β-xylanases, MxynB-8 showed excellent ability to hydrolyze hemicellulose of broadleaf plants, such as poplar. After 18 h, under 50°C, pH with the MxynB-8 dosage of 500 U/g, and substrate concentration of 2 mg/mL, the final XOS yield was 85.5%, and the content of XOS_2−3_ reached 93.9%. The enzymatic efficiency based on the poplar sawdust xylan in the raw material was 30.5%. Moreover, Xln-DT showed excellent sugar tolerant, which is applied as a candidate to apply in degradation of hemicellulose and the biotransformation of other natural active substances containing xylose. In addition, the process and enzymatic mode of poplar sawdust xylan with MxynB-8 and Xln-DT were investigated. The results showed that the enzymatic hydrolysis yield of poplar sawdust xylan was improved by adding Xln-DT, and the main product was d-xylose. The yield of enzymatic hydrolysis was higher when using MxynB-8 and Xln-DT together. This study provides a deep understanding of the double-enzyme combination hydrolytic conversion of wood polysaccharides to valuable products.

## Data Availability Statement

The datasets presented in this study can be found in online repositories. The names of the repository/repositories and accession number(s) can be found at: https://www.ncbi.nlm.nih.gov/, HQ385274; https://www.ncbi.nlm.nih.gov/, CP001146.1; https://www.ncbi.nlm.nih.gov/, WP_012547134.1; https://www.ncbi.nlm.nih.gov/, ACK42995.1.

## Author Contributions

QL, YJ, and XT: investigation. QL, LZ, and JP: supervision. QL: writing—original draft. QL and LZ: writing—review and editing. All authors have read and approved the final manuscript.

## Conflict of Interest

The authors declare that the research was conducted in the absence of any commercial or financial relationships that could be construed as a potential conflict of interest.

## References

[B1] Abbas N. Wright A. Pao L. Y. (2020). An update to the National Renewable Energy Laboratory baseline wind turbine controller. J. Phys. Conf. Ser. 1452:012002 10.1088/1742-6596/1452/1/012002

[B2] Azelee N. I. W. Jahim J. M. Ismail A. F. Fuzi S. F. Z. M. Rahman R. A. Illias R. M. (2016). High xylooligosaccharides (XOS) production from pretreated kenaf stem by enzyme mixture hydrolysis. Ind. Crops Products 81, 11–19. 10.1016/j.indcrop.2015.11.038

[B3] Belfaquih N. Jaspers C. Kurzatkowski W. Penninckx M. J. (2002). Properties of *Streptomyces* sp. endo-β-xylanases in relation to their applicability in kraft pulp bleaching. World J. Microbiol. Biotechnol. 18, 699–705. 10.1023/A:1016810018859

[B4] Bhalla A. Bischoff K. M. Sani R. K. (2014). Highly thermostable GH39 β-xylosidase from a *Geobacillus* sp. strain WSUCF1. BMC Biotechnol. 14:106. 10.1186/s12896-014-0106-8 25532585 PMC4300165

[B5] Blake A. D. Beri N. R. Guttman H. S. Cheng R. Gardner J. G. (2018). The complex physiology of *Cellvibrio japonicus* xylan degradation relies on a single cytoplasmic β-xylosidase for xylo-oligosaccharide utilization. Mol. Microbiol. 107, 610–622. 10.1111/mmi.13903 29266479

[B6] Chen M. X. Zhou H. Z. Zhu X. H. Tan Z. L Li X. D. (2015). Optimization of determination of protein in activated sludge by Bradford method. Environ. Sci. Technol. 38, 1–5.

[B7] Christersson L. (2008). Poplar plantations for paper and energy in the south of Sweden. Biomass Bioenergy 32, 997–1000. 10.1016/j.biombioe.2007.12.018

[B8] Collins T. Gerday C. Feller G. (2010). Xylanases, xylanase families and extremophilic xylanases. FEMS Microbiol. Rev. 29, 3–23. 10.1016/j.femsre.2004.06.005 15652973

[B9] Corrêa J. M. Graciano L. Abraho J. Loth E. A. Gandra R. F. Kadowaki M. K.. (2012). Expression and characterization of a GH39 β-xylosidase II from *Caulobacter crescentus*. Appl. Biochem. Biotechnol. 168, 2218–2229. 10.1007/s12010-012-9931-1 23054825

[B10] Dong L. H. Widagdo F. R. A. Xie L. F. Li F. R. (2020). Biomass and volume modeling along with carbon concentration variations of short-rotation poplar plantations. Forests 11:780 10.3390/f11070780

[B11] Georis J. Giannotta F. Buyl E. D. Granier B. Frere J. M. (2000). Purification and properties of three endo-β-1,4-xylanases produced by *Streptomyces* sp. strain S38 which differ in their ability to enhance the bleaching of kraft pulps. Enzyme Microb. Technol. 26, 178–186. 10.1016/S0141-0229(99)00141-6 10689075

[B12] Hsu C. K. Liao J. W. Chung Y. C. Hsieh C. P. Chan Y. C. (2004). Xylooligosaccharides and fructooligosaccharides affect the intestinal microbiota and precancerous colonic lesion development in rats. J. Nutr. 134, 1523–1528. 10.1093/jn/134.6.1523 15173423

[B13] Huang K. X. Das L. Guo J.M, Xu, Y. (2019). Catalytic valorization of hardwood for enhanced xylose-hydrolysate recovery and cellulose enzymatic efficiency via synergistic effect of Fe^3+^ and acetic acid. Biotechnol. Biofuels 12:248. 10.1186/s13068-019-1587-4 31636707 PMC6796388

[B14] Jacomini D. Bussler L. Corrêa J. M. Kadowaki M. K. Maller A. Silva J. L. D. C.. (2020). Cloning, expression and characterization of *C. crescentus*xyn A2 gene and application of Xylanase II in the deconstruction of plant biomass. Mol. Biol. Rep. 47, 4427–4438. 10.1007/s11033-020-05507-2 32424521

[B15] Jamaldheen S. B. Thakur A. Moholkar V. S. Goyal A. (2019). Enzymatic hydrolysis of hemicellulose from pretreated Finger millet (*Eleusine coracana*) straw by recombinant endo-1,4-β-xylanase and exo-1,4-β-xylosidase. Int. J. Biol. Macromol. 135, 1098–1106. 10.1016/j.ijbiomac.2019.06.010 31173827

[B16] Jun M. Lin W. F. Xu L. B. Liu S. H. Xue W. L. Chen S. F. (2020). Resistance to long-term bacterial biofilm formation based on hydrolysis-induced Zwitterion material with biodegradable and self-healing properties. Langmuir 36, 3251–3259. 10.1021/acs.langmuir.0c00006 32154728

[B17] Juturu V. Wu J. C. (2012). Microbial xylanases: engineering, production and industrial applications. Biotechnol. Adv. 30, 1219–1227. 10.1016/j.biotechadv.2011.11.006 22138412

[B18] Kholis M. N. Yopi Y. Meryandini A. (2015). Xylooligosaccharide production from Tobacco stalk xylan using xylanase *Streptomyces*sp. BO 3.2. Makara J. Sci. 19, 49–54. 10.7454/mss.v19i2.4738

[B19] Kiara W. Joann W. Alain C. David R. Lisa R. (2014). Soil carbon stocks in two hybrid poplar-hay crop systems in southern Quebec, Canada. Forests 5, 1952–1966. 10.3390/f5081952

[B20] Lahtinen M. H. Valoppi F. Juntti V. K. Heikkinen S. Mikkonen K. S. (2019). Lignin-rich PHWE hemicellulose extracts responsible for extended emulsion stabilization. Front. Chem. 7:871. 10.3389/fchem.2019.00871 31921786 PMC6927942

[B21] Li F. Yang S. Y. Zhao L. G. Pei J. J. (2012). Synonymous condon usage bias and overexpression of a synthetic *xynB* gene from *Aspergillus niger* NL-1 in *Pichia pastoris*. Bioresources 7, 2330–2343. 10.15376/biores.7.2.2330-2343

[B22] Li H. L. Xiong L. Chen X. D. Luo M. Chen X. F. Wang C. (2019a). Enhanced enzymatic hydrolysis of wheat straw via a combination of alkaline hydrogen peroxide and lithium chloride/*N,N*-dimethylacetamide pretreatment. Ind. Crops Products 137, 332–338. 10.1016/j.indcrop.2019.05.027

[B23] Li J. Zhang M. Wang D. H. (2019b). High-solids hydrolysis of corn stover to achieve high sugar yield and concentration through high xylan recovery from magnesium oxide-ethanol pretreatment. Bioresour. Technol. 302:122838. 10.1016/j.biortech.2020.122838 30780095

[B24] Li J. B. Feng P. Xiu H. J. Zhang M. Y. Li J. Y. Du M. Zhang X. F.. (2020a). Wheat straw components fractionation, with efficient delignification, by hydrothermal treatment followed by facilitated ethanol extraction. Bioresour. Technol. 316, 123882. 10.1016/j.biortech.2020.123882 32739576

[B25] Li M. Sun X. X. Chen Y. J. Shen T. Tan Z. T. Tang C. L. (2020b). Effect of xylan sulfate on the responsive swelling behavior of poly(methacrylatoethyl trimethyl ammonium chloride)-based composite hydrogels. Cellulose 27, 8745–8756. 10.1007/s10570-020-03402-4

[B26] Li Q. Chen M. Z. Zhao L. G. (2017). Cloning and expression of a thermophile GH11 xylanase gene and its application in xylooligosaccharide production. J. For. Eng. 2, 63–69. 10.13360/j.issn.2096-1359.2017.04.011

[B27] Li Q. Jiang Y. J. Tong X. Y. Pei J. J. Xiao W. Wang Z. Z.. (2020c). Cloning and characterization of the β-xylosidase from *Dictyoglomus turgidum* for high efficient biotransformation of 10-deacetyl-7-xylosltaxol. Bioorg. Chem. 94:103357. 10.1016/j.bioorg.2019.103357 31668798

[B28] Li Q. Wu T. Duan Y. W. Pei J. J. Zhao L. G. (2019c). Improving the thermostability and pH stability of *Aspergillus niger* xylanase by site-directed mutagenesis. Appl. Biochem. Microbiol. 55, 136–144. 10.1134/S0003683819020108

[B29] Li Q. Wu T. Qi Z. P. Zhao L.G Pei J. J. Tang F. (2018). Characterization of a novel thermostable and xylose-tolerant GH 39 β-xylosidase from *Dictyoglomus thermophilum*. BMC Biotechnol. 18:29. 10.1186/s12896-018-0440-3 29783967 PMC5963010

[B30] Liu Y. Li R. Wang J. Zhang X. H. Jia R. Gao Y.. (2017). Increased enzymatic hydrolysis of sugarcanebagasse by a novel glucose-and xylose-stimulated β-glucosidase from *Anoxybacillus flavithermus* subsp. *yunnanensis* E13^T^. BMC Biochem. 18:4. 10.1186/s12858-017-0079-z 28302049 PMC5356265

[B31] Menezes C. R. D. Silva I. S. Pavarina E. C. Dias E. F. G. Dias F. G. Grossman M. J. (2009). Production of xylooligosaccharides from enzymatic hydrolysis of xylan by the white-rot fungi Pleurotus. Int. Biodeterior. Biodegrad. 63, 673–678. 10.1016/j.ibiod.2009.02.008

[B32] Oliveira S. M. D. Perez S. M. Terrasan C. R. F. Fernandez M. R. Vieira M. F. Guisan J. M. (2018). Covalent immobilization-stabilization of β-1,4-endoxylanases from *Trichoderma reesei*: production of xylooligosaccharides. Process Biochem. 64, 170–176. 10.1016/j.procbio.2017.09.018

[B33] Ordomsky V. Khodakov A. Nijhuis T. A. Schouten J. C. (2015). Heterogeneously catalyzed reactive extraction for biomass valorization into chemicals and fuels. Green Process. Synth. 4, 369–377. 10.1515/gps-2015-0037

[B34] Qi C. S. Hou S. Y. Lu J. X. Xue W. W. Sun K. (2020). Thermal characteristics of birch and its cellulose and hemicelluloses isolated by alkaline solution. Holzforschung 74, 1099–1112. 10.1515/hf-2019-0285

[B35] Rohman A. Dijkstra B. W. Puspaningsih N. N. T. (2019). β-Xylosidases: structural diversity, catalytic mechanism, and inhibition by monosaccharides. Int. J. Mol. Sci. 20:5524. 10.3390/ijms20225524 31698702 PMC6887791

[B36] Sderling E. Pienihkkinen K. (2020). Effects of xylitol and erythritol consumption on mutans streptococci and the oral microbiota: a systematic review. Acta Odontol. Scand. 78, 599–608. 10.1080/00016357.2020.1788721 32633595

[B37] Sepulchro A. G. V. Pellegrini V. O. A. Briganti L. Araujo E. A. D. Araujo S. S. D. Polikarpov I. (2020). Transformation of xylan into value-added biocommodities using *Thermobacillus* composti GH10 xylanase. Carbohyd. Polym. 247:116714. 10.1016/j.carbpol.2020.116714 32829841

[B38] Shi H. Li X. Gu H. X. Zhang Y. Huang Y. J. Wang L. L.. (2013). Biochemical properties of a novel thermostable and highly xylose-tolerant β-xylosidase/α-arabinosidase from *Thermotoga thermarum*. Biotechnol. Biofuels 6:27. 10.1186/1754-6834-6-27 23422003 PMC3621209

[B39] Shi Z. L. Gong W. L. Zhang L. L. Dai L. Chen G. J. Wang L. S. (2018). Integrated functional-omics analysis of *Thermomyces lanuginosus*reveals its potential for simultaneous production of xylanase and substituted xylooligosaccharides. Appl. Biochem. Biotechnol. 187, 1515–1538. 10.1007/s12010-018-2873-5 30267287

[B40] Sun S. L. Wen J. L. Ma M. G. Sun R. C. (2013). Successive alkali extraction and structural characterization of hemicelluloses from sweet sorghum stem. Carbohyd. Polym. 92, 2224–2231. 10.1016/j.carbpol.2012.11.098 23399281

[B41] Tong X. Y. Li Q. Chen W. Q. Zhao L. G. (2020). Alkali extraction of xylan from poplar sawdust and preparation of xylooligosaccharide by enzymatic hydrolysis. J. For. Eng. 5, 61–68. 10.13360/j.issn.2096-1359.201904030

[B42] Vázquez M. J. Alonso J. L. Dominguez H. Parajó J. C. (2000). Xylooligosaccharides: manufacture and applications. Trends Food Sci. Technol. 11, 387–393. 10.1016/S0924-2244(01)00031-0

[B43] Vincent L. Hemalatha G. R. Elodie D. Coutinho P. M. Bernard H. (2014). The carbohydrate-active enzymes database (CAZy) in 2013. Nucleic Acids Res. 42, 490–495. 10.1093/nar/gkt1178 24270786 PMC3965031

[B44] Wang S.S Zou C. Yang H. P. Lou C. Cheng S. Z. Chao P. Wang C.. (2020). Effects of cellulose, hemicellulose, and lignin on the combustion behaviours of biomass under various oxygen concentrations. Bioresour. Technol. 320:124375. 10.1016/j.biortech.2020.124375 33186802

[B45] Wang Y. T. Fang Z. (2020). Catalytic biomass to renewable biofuels and biomaterials. Catalysts 10:480 10.3390/catal10050480

[B46] Wei H. Chen X. W. Joseph S. Erik K. Wang W. Ji Y. (2018). Kinetic modelling and experimental studies for the effects of Fe^2+^ ions on xylan hydrolysis with dilute-acid pretreatment and subsequent enzymatic hydrolysis. Catalysts 8:39 10.3390/catal8010039

[B47] Wen P. Y. Zhang T. Wang J. Y. Lian Z. N Zhang J. H. (2019). Production of xylooligosaccharides and monosaccharides from poplar by a two-step acetic acid and peroxide/acetic acid pretreatment. Biotechnol. Biofuels 12:87. 10.1186/s13068-019-1423-x 31011370 PMC6463647

[B48] Xian L. Li Z. Tang A. X. Qin Y. M. Li Q. Y. Liu H. B.. (2019). A novel neutral and thermophilic endoxylanase from *Streptomyces ipomoeae* efficiently produced xylobiose from agricultural and forestry residues. Bioresour. Technol. 285:121293. 10.1016/j.biortech.2019.03.132 30999191

[B49] Xu B. Dai L. M. Li J. J. Deng M. Miao H. B. Zhou J. P.. (2016). Molecular and biochemical characterization of a novel xylanase from *Massilia* sp. RBM26 isolated from thefeces of *Rhinopithecus* bieti. J. Microbiol. Biotechnol. 26, 9–19. 10.4014/jmb.1504.04021 26387816

[B50] Yan X. W. Lin C. Zhang L. Wang F. Chen Q. W. (2015). Analysis on chemical components changes in preparation process of cellulosic ethanol from poplar wood. J. Central South Univ. For. Technol. 35, 119–122. 10.14067/j.cnki.1673-923x.2015.02.023

[B51] Yang J. An X. Liu L. Tang S. Liu H. (2020). Cellulose, hemicellulose, lignin, and their derivatives as multi-components of bio-based feedstocks for 3D printing. Carbohyd. Polym. 250:116881. 10.1016/j.carbpol.2020.116881 33049824

[B52] Yang W. X. Bai Y. G. Yang P. L. Luo H. Y. Huang H. Q. Meng K.. (2015). A novel bifunctional GH51 exo-α-l-arabinofuranosidase/endo-xylanase from *Alicyclobacillus*sp. A4 with significant biomass-degrading capacity. Biotechnol. Biofuels 8:197. 10.1186/s13068-015-0366-0 26628911 PMC4666033

[B53] Yang X. Shi P. Huang H. Luo H. Wang Y. Zhang W.. (2014). Two xylose-tolerant GH43 bifunctional β-xylosidase/α-arabinosidases and one GH11 xylanase from Humicola insolens and their synergy in the degradation of xylan. Food Chem. 148, 381–387. 10.1016/j.foodchem.2013.10.062 24262572

[B54] Yang X. Shi P. Ma R. Luo H. Huang H. Yang P.. (2015). A new GH43 α-arabinofuranosidase from *Humicola insolens* Y1: biochemical characterization and synergistic action with a xylanase on xylan degradation. Appl. Biochem. Biotechnol. 175, 1960–1970. 10.1007/s12010-014-1416-y 25432346

[B55] Ye Y. Li X. Zhao J. (2017). Production and characteristics of a novel xylose-and alkali-tolerant GH43 β-xylosidase from *Penicillium oxalicum* for promoting hemicellulose degradation. Sci. Rep. 7:11600. 10.1038/s41598-017-11573-7 28912429 PMC5599605

[B56] Zhang R. Li N. Xu S. Han X. Li C. Wei X.. (2019a). Glycoside hydrolase family 39 β-xylosidases exhibit β-1,2-xylosidase activity for transformation of notoginsenosides: a new EC subsubclass. J. Agric. Food Chem. 67, 3220–3228. 10.1021/acs.jafc.9b00027 30834749

[B57] Zhang S. S. Xie J. C. Zhao L. G. Pei J. J. Su E. Z. Xiao W.. (2019b). Cloning, overexpression and characterization of a thermostable β-xylosidase from *Thermotoga petrophila* and cooperated transformation of ginsenoside extract to ginsenoside 20(S)-Rg3 with a β-glucosidase. Bioorg. Chem. 85, 159–167. 10.1016/j.bioorg.2018.12.026 30616097

[B58] Zhang Y. Bi P. Wang J. Jiang P. Wu X. Xue H. (2015). Production of jet and diesel biofuels from renewable lignocellulosic biomass. Appl. Energy 150, 128–137. 10.1016/j.apenergy.2015.04.023

[B59] Zhuo R. Yu H. B. Qin X. Ni H. X. Jiang Z. (2018). Heterologous expression and characterization of a xylanase and xylosidase from white rot fungi and their application in synergistic hydrolysis of lignocellulose. Chemosphere 212, 24–33. 10.1016/j.chemosphere.2018.08.062 30138852

