# Supplementary material for: Co-production of Xylooligosaccharides and Xylose From Poplar Sawdust by Recombinant Endo-1,4-β-Xylanase and β-Xylosidase Mixture Hydrolysis
Source: Front Bioeng Biotechnol. 2021 Feb 1;8:637397. doi: 10.3389/fbioe.2020.637397 (PMC7882696; doi:10.3389/fbioe.2020.637397)
Supplement: Supplementary file 1 [file Data_Sheet_1.docx]

**Fig.s1 HPLC analysis of xylan content in poplar sawdust after acid hydrolysis**


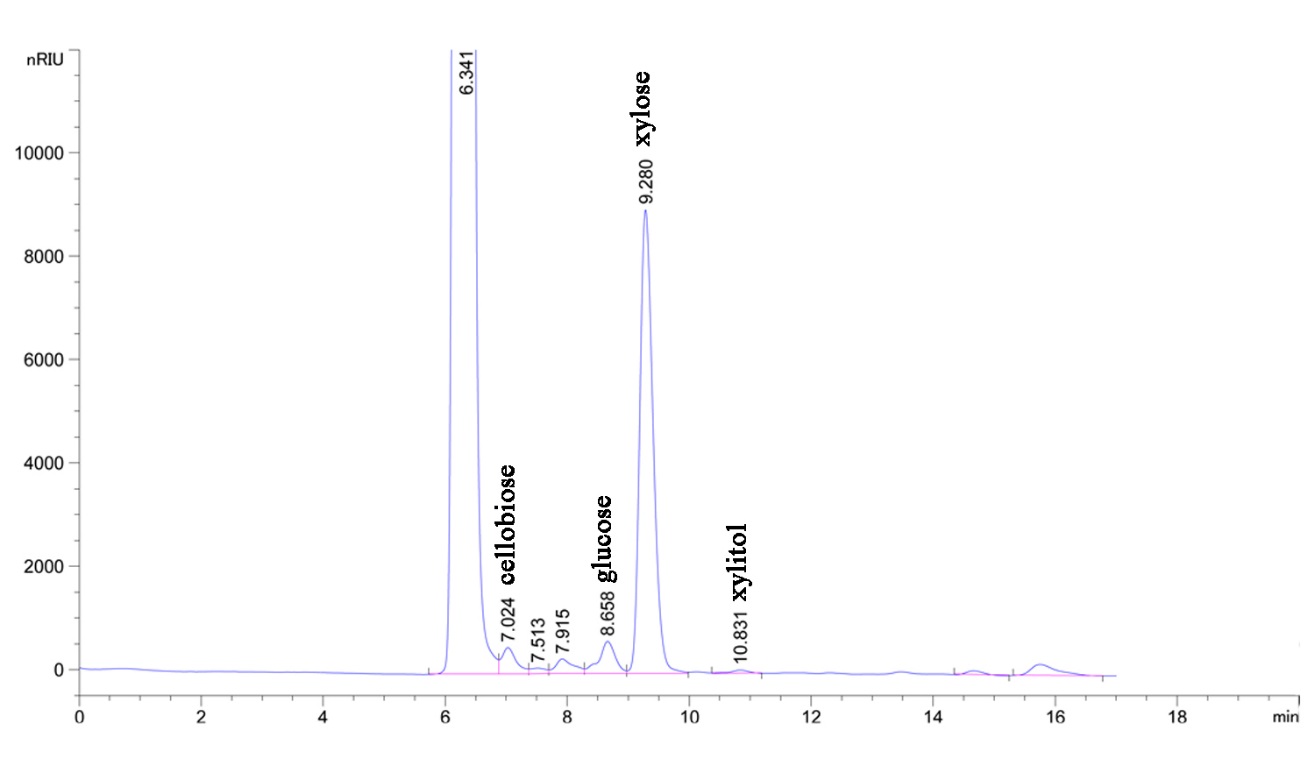


**Fig.s2 Infrared spectrum of poplar sawdust xylan**
